# The first week matters: App-based PROM trajectories and follow-up retention after endoscopic lumbar surgery

**DOI:** 10.1016/j.bas.2026.106080

**Published:** 2026-05-10

**Authors:** Phil Bohlen, Jannik Leyendecker, Cathryn Payne, Paula Krause, Eliana Bieler, Jan Bredow, Peer Eysel, Albert Telfeian, Peter Derman, Osama Kashlan, Sanjay Konakondla, John Ogunlade, Saqib Hasan, Meng Huang, Mark Mahan, Imad Khan, Raymond J. Gardocki, Mark Lambrechts, Anubhav Amin, David Huie, Dan Hafez, Galal Elsayed, Gregory Basil, Christoph P. Hofstetter, Endoscopic Spine Research Group (ESRG)

**Affiliations:** a Department of Neurological Surgery, University of Washington, Seattle, WA, USA; b Faculty of Medicine and University Hospital Cologne, University of Cologne, Department of Orthopedics and Trauma Surgery, Cologne, Germany; c Department of Orthopedics and Trauma Surgery, Krankenhaus Porz am Rhein, University of Cologne, Germany; d Department of Neurosurgery, Warren Alpert Medical School, Brown University, Providence, RI, USA; e Texas Back Institute, Plano, TX, USA; f Department of Neurosurgery, University of Michigan, Ann Arbor, Michigan, USA; g Department of Neurosurgery, Geisinger Neuroscience Institute, Danville, PA, USA; h Department of Neurological Surgery, Washington University School of Medicine, St. Louis, Missouri, USA; i Golden State Orthopedics & Spine, California, USA; j Houston Methodist Department of Neurosurgery, Texas, USA; k University of Utah NeuroSpine, Utah, USA; l Department of Orthopaedic Surgery, Vanderbilt Health, Tennesse, USA; m Department of Orthopaedic Surgery, Washington University in St. Louis, Missouri, USA; n Duke Spine Division, Duke University School of Medicine, North Carolina, USA; o Department of Neurological Surgery, Weill Cornell Medicine, New York, USA; p Department of Neurosurgery, University of Miami, USA

**Keywords:** Endoscopic spine surgery, Degenerative spine disease, Patient-reported outcome measures, Digital follow-up, Postoperative recovery

## Abstract

**Introduction:**

Full-endoscopic spine surgery (FESS) is increasingly utilized for the treatment of degenerative lumbar spinal disorders, with the aim of minimizing tissue disruption and facilitating recovery. Concurrently, postoperative follow-up is shifting toward digital platforms, enabling high-frequency collection of patient-reported outcome measures (PROMs). The characteristics of postoperative recovery trajectories and the determinants of long-term follow-up adherence in this setting remain incompletely defined.

**Research question:**

Do postoperative PROM trajectories and follow-up retention differ between full-endoscopic lumbar discectomy and decompression, and does early postoperative engagement predict long-term follow-up compliance?

**Material and methods:**

This prospective multicenter cohort study included adult patients undergoing lumbar full-endoscopic discectomy or decompression for degenerative pathology between 2018 and 2025. Postoperative PROMs were collected using a smartphone-based application on a daily basis during the first postoperative week and at predefined intervals up to six months. Outcomes included visual analog scale (VAS) scores for back and leg pain and the Oswestry Disability Index (ODI). Longitudinal PROM trajectories were analyzed, and follow-up retention was assessed using Kaplan–Meier analysis and Cox proportional hazards regression in a baseline-engaged cohort. Early postoperative PROM adherence was evaluated as a predictor of long-term retention.

**Results:**

A total of 478 patients were analyzed (279 discectomy, 199 decompression). Both cohorts demonstrated significant and sustained reductions in back and leg pain beginning on postoperative day one and persisting through six months (all p < 0.001). Functional recovery, as measured by ODI, followed a delayed course and exhibited a transient early postoperative increase in the decompression cohort. Six-month complete PROM datasets were available in 31.6% of discectomy patients and 48.4% of decompression patients. Follow-up retention differed significantly between procedures, with decompression associated with a lower hazard of dropout (adjusted HR 0.60, 95% CI 0.50–0.72). Higher PROM completion during the first postoperative week was independently associated with long-term follow-up compliance.

**Discussion:**

High-frequency app-based PROM assessment allows detailed evaluation of early and mid-term recovery following FESS. However, long-term outcome interpretation is constrained by follow-up attrition, which varies by procedure type.

**Conclusion:**

Early postoperative engagement appears to be a key determinant of durable follow-up and represents a potential target for improving longitudinal outcome assessment.

## Highlights

• App-based PROMs captured rapid and durable pain improvement after endoscopic lumbar surgery.
• Functional recovery followed a delayed and procedure-specific postoperative trajectory.
• PROM retention was higher after decompression than after discectomy.
• Early PROM completion predicted sustained 6-month follow-up compliance.
• The first postoperative week represents a key window to improve long-term follow-up.

## 1 Introduction

Low back pain is among the leading causes of disability worldwide and affects a substantial proportion of the general population, with a reported lifetime incidence of 65–85% (The global burden of tuberculosis, 2018). Although integrated multimodal strategies are recommended for its management (Müller-et al., 2017), outcomes are less favorable when low back pain is accompanied by radicular lower extremity symptoms caused by impingement of neural elements (Kongsted et al., 2013). Lumbar disc herniation and degenerative spinal canal stenosis represent the most common structural etiologies for radiculopathy and remain the predominant indications for surgical intervention in the lumbar spine (Grotle et al., 2019). Accordingly, interlaminar decompression and discectomy are among the most frequently performed spinal procedures, reflecting their established effectiveness and acceptable safety profile (Gadjradj et al., 2017).

At the population level, elective spine surgery has become a growing contributor to healthcare utilization and cost, with procedural volumes projected to continue increasing over the coming decades (Heck et al., 2023). This expansion in surgical demand has occurred in parallel with shifting patient expectations toward faster recovery and earlier return to function, while the patients themselves have become older and increasingly burdened by medical comorbidities and frailty. In this setting, there have been ongoing efforts to reduce perioperative morbidity and tissue disruption without compromising the effectiveness of neural decompression. These pressures have driven the progressive refinement of minimally invasive surgical techniques, including the development of full-endoscopic spine surgery (FESS) as an alternative to conventional open and microsurgical approaches (Ruetten et al., 2008). The transition toward minimally invasive and endoscopic spine surgery has implications at both the healthcare system and individual patient levels. Reductions in hospital length of stay and resource utilization may translate into societal economic benefits and increased institutional capacity (Gadjradj et al., 2022). Concurrently, less invasive surgical strategies have been associated with improved patient engagement and adherence to postoperative care pathways, a trend observed across multiple surgical disciplines (Basil and Wang, 2019; Pendharkar et al., 2018). At the same time, patients increasingly expect precise and reliable counseling regarding postoperative recovery. As the spectrum of minimally invasive techniques continues to expand and rapid rehabilitation is frequently emphasized, patients are often required to choose between surgical approaches based on anticipated recovery trajectories (Makoul and Clayman, 2006; Derman et al., 2025). However, such expectations are commonly shaped by expert opinion rather than high-quality longitudinal data, resulting in uncertainty regarding realistic postoperative outcomes following FESS.

The early postoperative phase remains critical for the detection of complications, facilitation of mobilization, and overall patient satisfaction, underscoring the importance of structured postoperative follow-up in spine surgery. Unlike other surgical specialties that rely on routine physical examinations to detect and mitigate postoperative adverse events, follow-up after FESS is predominantly guided by clinical assessment and patient-reported outcomes rather than invasive testing (Leyendecker et al., 2024a). Consequently, postoperative monitoring following FESS can be accomplished without in person clinic visits (Derman et al., 2025). In this context, digital, app-based solutions have emerged to support postoperative care by enabling remote, high-frequency assessment of symptoms and functional recovery replacing scheduled clinic visits. Prior studies have demonstrated that such approaches may improve follow-up adherence while reducing in-person postoperative visits, with high levels of patient satisfaction (Leyendecker et al., 2025).

Beyond facilitating clinical surveillance, continuous remote data acquisition allows for detailed characterization of postoperative pain and recovery trajectories that are not captured through routine clinical follow-up visits. However, sustaining patient engagement over extended postoperative intervals remains a key limitation of digital patient-reported outcome measure collection, particularly beyond the early recovery phase. Early postoperative engagement may influence patient long-term follow-up compliance, particularly in digitally monitored cohorts. Accordingly, the present study aimed to characterize digitally collected postoperative PROM trajectories following full-endoscopic lumbar discectomy and decompression in a large prospective cohort with follow-up extending to six months. In addition, we sought to identify patient- and treatment-related factors associated with sustained compliance to app-based postoperative follow-up after FESS, with particular emphasis on early postoperative adherence.

## 2 Material and methods

### 2.1 Study design and patient population

This multicenter study was conducted across the United States and included English-speaking patients aged 18 years or older. Patients were eligible if they underwent lumbar FESS for degenerative spinal pathology between January 2018 and December 2025. All participating surgeons are highly experienced in full-endoscopic techniques. Data collection was performed prospectively at participating centers, each of which obtained approval from its local institutional review board (IRB). Full-endoscopic spine surgery was defined as a procedure performed using a uniportal working-channel endoscope incorporating a light source, camera, and continuous irrigation system (Hofstetter et al., 2020; Schmidt et al., 2020).

### 2.2 Digital follow-up platform and data collection

Postoperative follow-up and data collection were conducted using the SPINEhealthie smartphone application (Prasse et al., 2023). Enrollment required access to a compatible smartphone and the ability to use the application. SPINEhealthie is a standardized digital platform designed to facilitate remote postoperative follow-up and longitudinal data acquisition. Patient-reported inputs are transmitted through the application, enabling continuous symptom monitoring and asynchronous patient–provider communication within the postoperative care workflow. The platform collects baseline demographic and clinical variables, including age, sex, body mass index (BMI), employment status, comorbidities, and surgical details, as well as perioperative and postoperative complications. Anesthesia type was recorded as part of the surgical details. De-identified patient data were aggregated across all participating centers following IRB approval, in accordance with national regulatory requirements and the principles of the 1975 Declaration of Helsinki. Patient-reported outcome measures (PROMs) were collected daily during the first seven postoperative days and at prespecified follow-up intervals, including 2 weeks, 3 months, 6 months, and 1 year after surgery (Pan et al., 2023). The PROMs included the VAS for back and leg pain and the ODI (v2.1a), a validated measure of back-related functional disability (Fairbank and Pynsent, 2000).

### 2.3 Surgical subgroups

The study cohort was stratified into two groups based on the primary surgical intervention: full-endoscopic lumbar discectomy and full-endoscopic lumbar decompression. Patients presenting with acute or subacute lumbar disc herniation associated with radicular symptoms concordant with imaging findings were assigned to the discectomy group. Patients with other degenerative pathologies resulting in central, lateral recess, or foraminal stenosis were allocated to the decompression group. This classification was chosen to ensure methodological consistency across outcome analyses. No further subgrouping by specific endoscopic technique was performed was used for the primary analyses. Within the discectomy cohort EELD represented a rare variant and was addressed in a separate sensitivity analysis (Table S1). To address heterogeneity within the decompression cohort, we performed a sensitivity analysis (Tables S2.1-2) comparing decompression of the spinal canal (LE-ULBD, IE-LRD) and foraminal decompression (ICELF, TELF, TE-LRD).

### 2.4 PROM trajectory and follow-up compliance analysis

For PROM trajectory analyses, patients were excluded if PROM submissions were missing for the pre-operative baseline, during the first postoperative week, or at the 3-month follow-up. To evaluate loss to follow-up, a baseline-engaged cohort was defined as patients with complete preoperative PROM data. PROM retention was operationalized as the time to the first missed scheduled follow-up within predefined assessment windows (postoperative days 1–7, 3 months, and 6 months). Patients were considered retained as long as all eligible scheduled follow-up assessments were completed. The early postoperative window (days 1–7) was considered complete if at least one PROM submission occurred within this interval. The Patients of both cohorts were also dichotomized into two predefined early-engagement cohorts based on the number of complete PROMs to analyze the six-month-completion probability.: Completed PROMs on the postoperative days 1-3 versus 4-7 complete PROM days in the first postoperative week.

### 2.5 Statistical analysis

All statistical analyses and graphical visualizations were performed using R (version 4.5.2). Patients were categorized into discectomy and decompression groups as defined above. Continuous variables are presented as means ± standard deviation, and categorical variables are reported as counts and proportions (%). Between-group comparisons of continuous variables were conducted using Welch's two-sample *t*-test, while categorical variables were compared using Pearson's chi-square test. A two-sided *p* value < 0.05 was considered statistically significant. Within-group longitudinal comparisons were performed for each postoperative time point relative to preoperative baseline PROM values. Estimated marginal means were calculated using Dunnett's adjustment for multiple comparisons, with two-sided *p* values reported. Statistical significance was defined as *p* < 0.05. Graphical visualization of PROM trajectories was performed using line plots with 95% confidence bands. For analyses of PROM retention, follow-up time was administratively censored at the database lock date. A time-to-event variable was defined as the first eligible follow-up at which PROMs were not submitted. Patients who had not yet reached a given follow-up window by the lock date were treated as not eligible for that time point and were censored accordingly. Kaplan–Meier curves were generated to compare PROM retention between groups, and differences were assessed using the log-rank test. To estimate hazard ratios (HRs) for loss of PROM retention, Cox proportional hazards regression models were constructed using the Efron method for ties. Models included procedure group, age (per 10-year increment), sex, and baseline PROMs, and were stratified by employment status and age category (<50 years, 50–64 years, >65 years). Given the between-group difference in symptom duration, we performed an additional sensitivity Cox model adjusting for symptom duration. The proportional hazards assumption was assessed using Schoenfeld residuals.

## 3 Results

### 3.1 Patient cohort and baseline characteristics

A total of 478 patients were included in the study cohort, of whom 279 underwent full-endoscopic lumbar discectomy and 199 underwent full-endoscopic lumbar decompression. The mean age was 51.23 ± 15.24 years in the discectomy group compared with 62.72 ± 12.64 years in the decompression group, representing a statistically significant difference (p < 0.001). The two cohorts also differed with respect to sex distribution, with female patients comprising 49.5% of the discectomy group and 34.7% of the decompression group (Table 1). Mean body mass index was similar between groups (29.66 ± 7.24 in the discectomy group vs. 30.22 ± 7.10 in the decompression group; p = 0.799). Pain duration was shorter in the Discectomy group than in the Decompression group (12.2 ± 17.5 vs 28.5 ± 41.7, p < 0.001).

**Table 1:** Demographics of the patient population.

Variable	Discectomy (n = 279)	Decompression (n = 199)	p-value
Age, yrs	51.23 ± 15.24	62.72 ± 12.64	**<0.001**
Sex			**0.002**
Female	138 (49.5)	69 (34.7)	
Male	141 (50.5)	130 (65.3)	
BMI	29.66 ± 7.24	30.22 ± 7.10	0.406
Pain duration, mos	12.2 ± 17.5	28.5 ± 41.7	**<0.001**
Comorbidities			
Hypertension	66 (45.5)	80 (62.0)	**0.009**
Hyperlipidemia	13 (9.0)	13 (10.1)	0.915
Diabetes mellitus	34 (23.4)	25 (19.4)	0.503
Coronary artery disease	6 (4.1)	5 (3.9)	>0.999
Asthma/COPD	18 (12.4)	19 (14.7)	0.702
Arthritis	40 (27.6)	40 (31.0)	0.625
Employment status			**<0.001**
Employed	197 (72.2)	86 (43.4)	
Not employed	18 (6.6)	13 (6.6)	
Retired	41 (15.0)	80 (40.4)	
Disabled	17 (6.2)	19 (9.6)	
Surgical procedure			**<0.001**
TELD	151 (54.1)	0 (0.0)	
IELD	125 (44.8)	0 (0.0)	
EELD	3 (1.1)	0 (0.0)	
LE-ULBD	0 (0.0)	132 (66.3)	
TELF	0 (0.0)	31 (15.6)	
IE-LRD	0 (0.0)	30 (15.1)	
ICELF	0 (0.0)	5 (2.5)	
TE-LRD	0 (0.0)	1 (0.5)	
Surgery duration per level, mins	70.81 ± 41.23	93.70 ± 39.48	**<0.001**
Blood loss per level, ml	5.44 ± 4.52	7.65 ± 10.32	**0.005**
No. of operated levels			**<0.001**
1	265 (95.0)	156 (78.4)	
2	14 (5.0)	37 (18.6)	
	0 (0.0)	6 (3.0)	
2-wk follow-up wound image	262 (93.9)	188 (94.5)	0.951
Wound-related issues			
Fever	3/262 (1.1)	1/188 (0.5)	0.644
Night sweats	26/262 (9.9)	15/188 (8.0)	0.588
Chills	9/262 (3.4)	3/188 (1.6)	0.369
Wound drainage	4/262 (1.5)	0/188 (0.0)	0.144
Redness	20/262 (7.6)	10/188 (5.3)	0.436
Tenderness	37/262 (14.1)	15/188 (8.0)	0.063
Dural tear	7 (2.9)	15 (9.0)	**0.014**

Values are depicted as mean ± SD or absolute number (%) unless otherwise indicated.

Boldface type indicates statistical significance.

COPD = chronic obstructive pulmonary disease; TELD = transforaminal endoscopic lumbar discectomy; IELD = interlaminar endoscopic lumbar discectomy; EELD = extraforaminal endoscopic lumbar discectomy; LE-ULBD = lumbar endoscopic unilateral laminotomy; TELF = transforaminal endoscopic lumbar foraminotomy; IE-LRD = interlaminar endoscopic lateral recess decompression; ICELF = interlaminar contralateral endoscopic lumbar foraminotomy; TE-LRD = transforaminal full-endoscopic lateral recess decompression.

Employment status at the time of surgery differed significantly between cohorts, with 72.2% of patients in the discectomy group employed compared with 43.4% in the decompression group. Conversely, a higher proportion of patients in the decompression group were retired (40.4% vs. 15.0%; p < 0.001). The majority of patients in both cohorts underwent single-level procedures, although this was more common in the discectomy group (95.0% vs. 78.4%; p < 0.001; Table 1). Most procedures were performed under general anesthesia (discectomy: 224/279; decompression:196/199). Restriction to general anesthesia cases did not change early PROM trajectories (Table S3).

### 3.2 Surgery related issues and follow-up

Most of the patients in both cohorts submitted an image of the surgical wound at 14 days postoperatively (93.9% in the discectomy group vs. 94.5% in the decompression group; p = 0.951). The most frequently reported postoperative issues were night sweats and localized tenderness in both groups. A dural tear, a recognized complication of lumbar discectomy and decompression, occurred in 7 patients in the discectomy group and in 15 patients in the decompression group, representing a statistically significant difference between cohorts (p = 0.007; Table 1).

### 3.3 Discectomy group

**Fig. 1:**
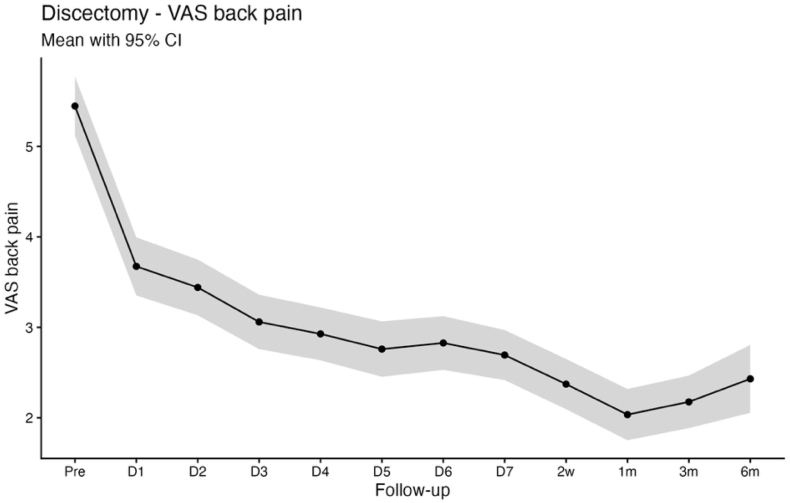
Line chart with confidence band depicting the mean VAS Back score for the discectomy group.

**Fig. 2:**
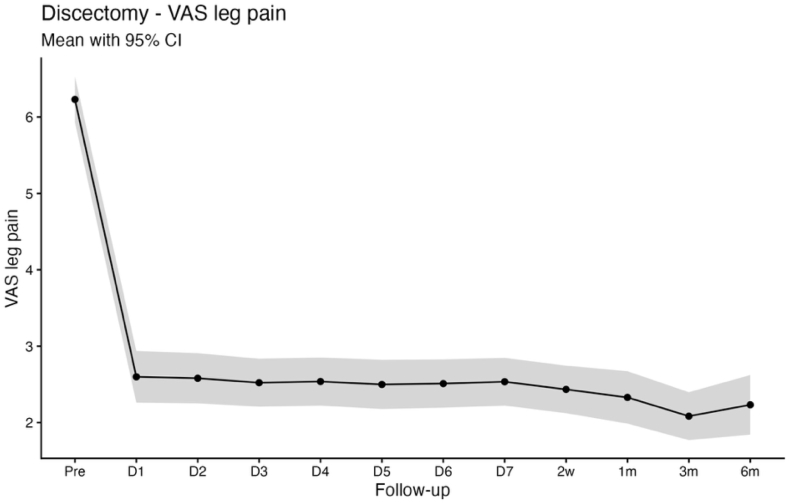
Line chart with confidence band depicting the mean VAS Leg score for the discectomy group.

**Fig. 3:**
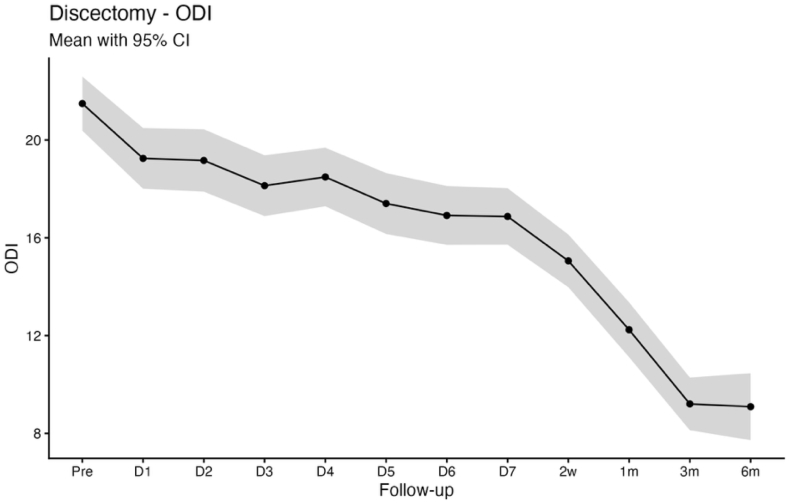
Line chart with confidence band depicting the mean ODI score for the discectomy group.

Baseline mean scores were 5.4 ± 2.8 for VAS back pain, 6.2 ± 2.5 for VAS leg pain, and 21.5 ± 9.4 for the Oswestry Disability Index (ODI). Statistically significant improvements compared with baseline were observed as early as postoperative day 1 and were sustained throughout the first postoperative week for back pain (3.7 ± 2.6; p < 0.001), leg pain (2.6 ± 2.8; p < 0.001), and ODI (19.3 ± 10.2; p < 0.001). At 6 months, patient-reported outcomes demonstrated durable improvement, with mean scores of 2.4 ± 2.6 for VAS back pain, 2.2 ± 2.6 for VAS leg pain, and 9.1 ± 9.4 for ODI (all p < 0.001; Table 2; Fig. 1, Fig. 2, Fig. 3).

**Table 2:** App-based transmitted patient outcomes for VAS back pain, VAS leg pain, and ODI at the preoperative time point, first 7 postoperative days, 2 weeks, 3 and 6 months.

		Preop	Day 1	Day 2	Day 3	Day 4	Day 5	Day 6	Day 7	2 Wks	3 Mos	6 Mos
Discectomy		n = 279	n = 260	n = 256	n = 252	n = 255	n = 244	n = 248	n = 261	n = 274	n = 279	n = 185
	VAS back pain	5.4 ± 2.8	3.7 ± 2.6; **< 0.001**	3.4 ± 2.5; **< 0.001**	3.0 ± 2.4; **< 0.001**	2.9 ± 2.4; **< 0.001**	2.8 ± 2.4; **< 0.001**	2.8 ± 2.4; **< 0.001**	2.7 ± 2.3; **< 0.001**	2.4 ± 2.3; **< 0.001**	2.2 ± 2.5; **< 0.001**	2.4 ± 2.6; **< 0.001**
	VAS leg pain	6.2 ± 2.5	2.6 ± 2.8; **< 0.001**	2.6 ± 2.7; **< 0.001**	2.5 ± 2.5; **< 0.001**	2.6 ± 2.6; **< 0.001**	2.5 ± 2.6; **< 0.001**	2.5 ± 2.5; **< 0.001**	2.6 ± 2.6; **< 0.001**	2.4 ± 2.6; **< 0.001**	2.1 ± 2.7; **< 0.001**	2.2 ± 2.6; **< 0.001**
	ODI	21.5 ± 9.4	19.3 ± 10.2; **< 0.001**	19.2 ± 10.4; **< 0.001**	18.1 ± 10.0; **< 0.001**	18.5 ± 9.8; **< 0.001**	17.4 ± 9.9; **< 0.001**	17.0 ± 9.7; **< 0.001**	16.9 ± 9.5; **< 0.001**	15.1 ± 9.1; **< 0.001**	9.2 ± 9.1; **< 0.001**	9.1 ± 9.4; **< 0.001**
Decompression		n = 199	n = 185	n = 191	n = 186	n = 183	n = 183	n = 181	n = 192	n = 197	n = 199	n = 140
	VAS back pain	5.6 ± 2.6	4.2 ± 2.6; **< 0.001**	4.3 ± 2.6; **< 0.001**	3.9 ± 2.6; **< 0.001**	3.5 ± 2.5; **< 0.001**	3.4 ± 2.4; **< 0.001**	3.1 ± 2.4; **< 0.001**	3.0 ± 2.4; **< 0.001**	2.7 ± 2.2; **< 0.001**	2.6 ± 2.7; **< 0.001**	2.8 ± 2.6; **< 0.001**
	VAS leg pain	6.0 ± 2.7	2.8 ± 2.7; **< 0.001**	3.2 ± 2.8; **< 0.001**	2.9 ± 2.7; **< 0.001**	3.0 ± 2.7; **< 0.001**	2.9 ± 2.6; **< 0.001**	2.8 ± 2.5; **< 0.001**	2.9 ± 2.6; **< 0.001**	2.8 ± 2.5; **< 0.001**	2.5 ± 2.8; **< 0.001**	2.8 ± 2.6; **< 0.001**
	ODI	19.5 ± 8.4	21.3 ± 10.0; **0.01392**	21.6 ± 10.6; **0.00424**	21.4 ± 10.4; **0.01308**	20.3 ± 10.3; 0.64144	19.6 ± 9.6; 0.99940	19.2 ± 9.4; 0.93906	19.3 ± 9.6; 0.99650	17.0 ± 9.2; **< 0.001**	10.8 ± 9.0; **< 0.001**	10.6 ± 9.0; **< 0.001**

Values are depicted as mean ± SD; p value. The p values refer to the reported value of the respective day compared with the preoperative value. Boldface type indicates statistical significance.

PROM trajectories remained robust after exclusion of rare technical variants (EELD), with no meaningful changes in early or longitudinal outcomes (Table S1).

### 3.4 Decompression group

**Fig. 4:**
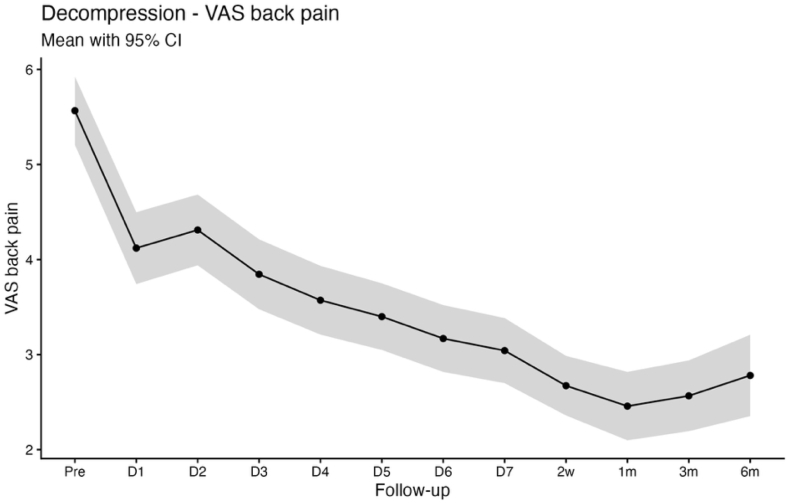
Line chart with confidence band depicting the mean VAS Back score for the decompression group.

**Fig. 5:**
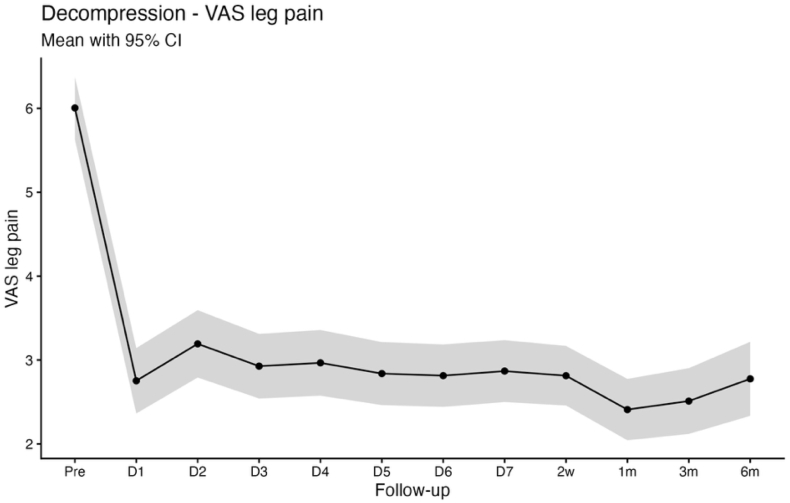
Line chart with confidence band depicting the mean VAS Leg score for the decompression group.

**Fig. 6:**
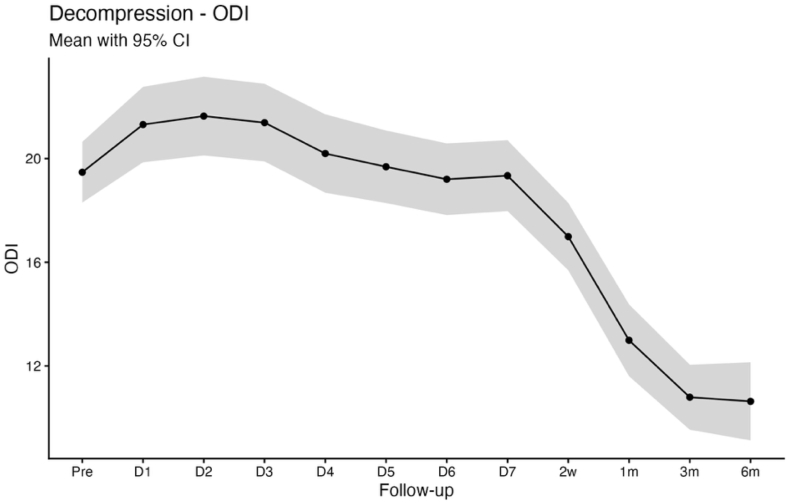
Line chart with confidence band depicting the mean ODI score for the decompression group.

Baseline PROMs in the decompression cohort were comparable to those observed in the discectomy group, with mean scores of 5.6 ± 2.6 for VAS back pain, 6.0 ± 2.7 for VAS leg pain, and 19.5 ± 8.4 for the ODI. A similar postoperative recovery pattern was observed for back and leg pain, with significant improvement relative to baseline evident from the first postoperative day (VAS back pain 4.2 ± 2.6; VAS leg pain 2.8 ± 2.7; both p < 0.001). These improvements were sustained through 6 months of follow-up (VAS back pain 2.8 ± 2.6; VAS leg pain 2.8 ± 2.6; both: p < 0.001). In contrast to the rapid reduction in pain scores, ODI demonstrated a biphasic postoperative trajectory, characterized by a transient increase during the early postoperative period (postoperative day 1: 21.3 ± 10.0, p = 0.017; day 2: 21.6 ± 10.6, p = 0.006; day 3: 21.4 ± 10.4, p = 0.017). A significant improvement was observed by 2 weeks postoperatively (17.0 ± 9.2; p < 0.001), with continued improvement at 3 and 6 months (3 months: 10.8 ± 9.0; 6 months: 10.6 ± 9.0; both p < 0.001 vs. Baseline; Table 2; Fig. 4, Fig. 5, Fig. 6).

Sensitivity analyses stratified by decompression strategy (spinal canal vs foraminal) showed no technique-dependent differences in PROM trajectories (Tables S2.1-2).

### 3.5 Retention and dropout patterns

**Fig. 7:**
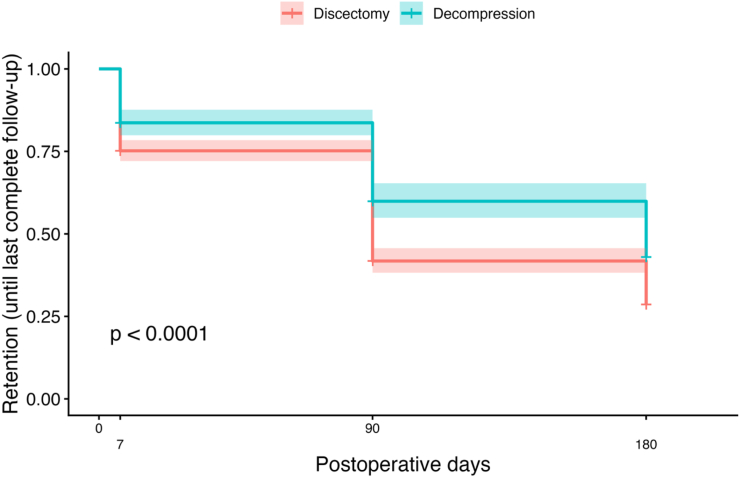
PROM retention through 6 months. Kaplan–Meier curves show retention of complete PROM follow-up among baseline-engaged patients eligible.

Among 1252 eligible patients undergoing full-endoscopic lumbar discectomy or decompression recorded in the database, 1058 (84.5%) constituted the baseline-engaged cohort with complete preoperative PROM data (discectomy *n* = 709; decompression *n* = 349). Of these, 929 patients were administratively eligible for six-month follow-up (discectomy *n* = 623; decompression *n* = 306). At the six-month time point, complete PROM datasets were available for 197 of 623 discectomy patients (31.6%) and for 148 of 306 decompression patients (48.4%). Kaplan–Meier analysis of PROM retention over 180 days demonstrated significantly lower retention in the discectomy group compared with the decompression group (log-rank χ^2^ = 25.6, p = 4.27 × 10^−7^). Median retention time was 90 days (95% CI 90–90) for discectomy and 180 days (95% CI 180–180) for decompression (Table 3; Fig. 7).

**Table 3:** Number at risk by group and time point.

Group	Day 0	Day 7	Day 90	Day 180
Decompression	349	349	278	195
Discectomy	709	709	502	270

Values represent the number of patients remaining at risk at each postoperative time point in the Kaplan–Meier analysis.

Confidence bands indicate 95% confidence intervals; p-value from the log-rank test.

### 3.6 Cox regression

In unadjusted Cox proportional hazards analysis using the Efron method for ties, decompression was associated with a lower hazard of loss of PROM retention compared with discectomy (HR 0.65, 95% CI 0.55–0.77; p < 0.001). This association persisted in the primary adjusted model, which was stratified by employment status and adjusted for age (per 10 years), sex, and baseline PROMs (adjusted HR 0.60, 95% CI 0.50–0.72; p < 0.001). Results remained consistent in a sensitivity model additionally stratified by age category (HR 0.61, 95% CI 0.51–0.73; p < 0.001). In a symptom duration-adjusted sensitivity model, decompression remained associated with a lower hazard of loss of PROM retention (HR 0.51, 95% CI 0.39-0.66, p < 0.001). The effect of treatment group on PROM retention was stable over time. Although age showed indications of time-dependent behavior in the primary adjusted model, findings were confirmed in sensitivity analyses stratified by age category (Table 4). Among baseline-engaged patients with at least one completed PROM during postoperative days 1–7 (n = 746), early postoperative engagement was strongly associated with six-month PROM completion. In the discectomy group, six-month completion was observed in 4 of 86 patients (4.7%) with low early engagement (1–3 of 7 days) compared with 181 of 396 patients (45.7%) with high early engagement (4–7 of 7 days). Similarly, in the decompression group, completion rates were 4 of 21 patients (19.0%) in the low-engagement cohort and 136 of 243 patients (56.0%) in the high-engagement cohort.

**Table 4:** Multivariable Cox proportional hazards regression for loss of PROM retention (time-to-dropout).

Variable	HR	95% CI	p-value
Group (Decompression vs Discectomy)	0.61	0.51-0.73	<0.001
Age (per 10 years)	1.08	1.01-1.14	0.019
Sex (Male vs Female)	1.17	1.00-1.36	0.056
Baseline back pain (VAS)	1.01	0.98-1.05	0.396
Baseline leg pain (VAS)	0.99	0.96-1.03	0.658
Baseline ODI	1.00	0.99-1.01	0.587

Cox proportional hazards model (Efron method for ties); stratified by employment status.

In logistic regression analyses, completion of PROMs on 4 - 7 of the first 7 postoperative days was associated with markedly higher odds of six-month completion (unadjusted OR 12.2, 95% CI 6.20–27.6; adjusted OR 11.2, 95% CI 5.66–25.5; both: p < 0.001). Consistently, Cox models evaluating PROM attrition demonstrated a substantially lower hazard of dropout in the high-engagement cohort compared with the low-engagement cohort (unadjusted HR 0.28, 95% CI 0.22–0.35; minimally adjusted for treatment group HR 0.29, 95% CI 0.23–0.37; both: p < 0.001).

## 4 Discussion

We observed a rapid and sustained postoperative improvement in pain following both endoscopic discectomy and decompression using high-frequency app-based PROM collection. However, interpretation of these recovery trajectories was closely linked to follow-up behavior. In the baseline-engaged cohort, PROM retention was higher after decompression than after discectomy, underscoring that procedure type and follow-up compliance should be considered jointly. Among patients eligible for 6-month follow-up, complete PROM datasets were available in 31.6% of discectomy patients and 48.4% of decompression patients, and time-to-dropout differed significantly between groups on Kaplan–Meier analysis, favoring decompression. Notably, patterns of early postoperative engagement emerged as an important determinant of sustained follow-up, highlighting the first postoperative week as a critical window influencing the interpretability of longer-term outcomes.

### 4.1 Interpretation of recovery trajectories 

Pain relief occurs quickly after decompression of compromised neural structures, which plausibly explains the rapid improvement in VAS back and leg pain in both cohorts, whereas functional recovery is influenced by additional factors including symptom chronicity, postoperative stiffness, fatigue, cautious movement, and deconditioning that may delay ODI improvement to later timepoints (Leyendecker et al., 2024b; Yeung et al., 2019). This dissociation between early pain relief and functional recovery is clinically relevant for counseling and for setting expectations in the early postoperative period (Grundnes et al., 2024). Notably, patients undergoing decompression typically present with long-standing neurogenic claudication and prolonged preoperative functional limitation, whereas discectomy patients more often have shorter symptom duration and acute radicular pain. Consistent with this distinction, ODI improved immediately following discectomy, while a biphasic ODI trajectory was observed in the decompression group, characterized by transient worsening during the first three postoperative days, normalization by postoperative days 4-7, and sustained improvement from week 2 through 6 months. An early postoperative increase in ODI should therefore not be interpreted as an indicator of unsuccessful recovery or impaired measurement validity, particularly when pain metrics improve concurrently, but rather as a reflection of short-term functional burden during early recovery. Seeing this pattern in a larger cohort is useful for clinicians, as it allows reassurance of patients who report early postoperative disability fluctuations despite rapid pain relief (Leyendecker et al., 2024b). This demonstrates that high-resolution app-based follow-up is informative beyond conventional clinical timepoints, where patients are typically assessed several weeks after surgery (Akoto et al., 2023). By assessing PROMs during the first postoperative week, app-based monitoring can reveal short-term postoperative disability changes that would otherwise be missed or misattributed (Shinwari et al., 2025). Because of the fact, that discectomy and decompression cohorts represent distinct patient populations, we report PROM trajectories within cohorts and interpret differences as associative rather than causal, with residual confounding remaining possible.

### 4.2 Digital follow-up feasibility and retention

Substantial attrition in PROM completion was observed even among baseline-engaged patients undergoing full-endoscopic spine surgery, underscoring the challenge of sustaining longitudinal follow-up despite digital data collection. After restricting the analysis to patients with sufficient administrative follow-up to allow a six-month observation window, fewer than half submitted a complete PROM set at six months. Retention was defined conservatively as completion of all three PROM instruments, prioritizing multidimensional outcome assessment but potentially underestimating partial engagement. Importantly, follow-up retention differed by procedure type: patients undergoing decompression demonstrated consistently higher PROM submission rates than those undergoing discectomy, with a significantly longer time to dropout on Kaplan-Meier analysis. Early postoperative PROM values and early symptom improvement did not differ meaningfully between completers and non-completers, suggesting that loss to follow-up was not driven solely by early clinical recovery but more likely reflects modifiable engagement- or workflow-related factors. Procedure-specific differences in retention may, in part, reflect differences in underlying indications and patient characteristics. In this cohort, patients undergoing decompression were older and more frequently retired, whereas discectomy patients were younger and more often employed. Such differences may influence time availability, competing demands, and the perceived value of continued outcome reporting. Patients with symptomatic spinal canal stenosis often experience a prolonged history of pain and gradual functional decline prior to surgery, a pattern also observed in the present cohort. These extended symptom trajectories may foster sustained motivation for postoperative reporting, whereas patients undergoing discectomy may experience more rapid symptom relief and disengage earlier from follow-up (Levy et al., 2023; Geere et al., 2025). Complementing these observations, early postoperative PROM adherence emerged as a strong predictor of long-term retention. Patients who completed PROMs on at least four of the first seven postoperative days demonstrated substantially higher six-month completion rates than those with only one to three completed days, an association that persisted after adjustment for treatment group. This pattern may reflect an early behavioral “imprinting” effect, whereby establishment of a reporting routine during the initial postoperative period promotes sustained engagement. Alternatively, intrinsic patient characteristics such as motivation, digital literacy, or health-related behaviors may influence both early and long-term adherence. Taken together, these findings suggest that the first postoperative week represents a practical and clinically relevant window for targeted retention interventions aimed at improving durable follow-up and enhancing the interpretability of longitudinal outcome data.

### 4.3 Clinical and implementation implications

We envision that enhancement of early postoperative engagement combined with targeted reminders could improve patient long-term follow-up compliance. In addition, integrating app-based PROM collection with routine clinical workflows may further improve compliance (Endler et al., 2020). Text message reminders have demonstrated to promote patient compliance in other clinical settings (Hofstetter et al., 2015). We have therefore implemented automated text message reminders in our follow-up workflow. Future studies are planned to compare retention metrics (e.g., time-to-dropout and complete PROM set completion) in the post-implementation cohort versus the current cohort.

## 5 Limitations

Several limitations should be acknowledged. The retention and PROM completion reflect reporting behavior and may be influenced by smartphone literacy, socioeconomic factors, notification settings, language, and other barriers unrelated to clinical recovery. Digital follow-up is inherently limited to patients with smartphone access, which varies across demographic strata, which may affect generalizability. Another factor contributing to diminishing retention is that our retention definition required complete submission of all three PROMs (VAS back, VAS leg, and ODI), representing a conservative standard that prioritizes multidimensional recovery assessment. Presenting any PROM or partial-completion sensitivity analyses could further contextualize these findings, as some patients may continue to report pain scores while discontinuing to more elaborated questionnaires.

The use of the ODI within the first postoperative week may be considered a limitation, as this instrument has not been separately validated as a stand-alone ultra-early postoperative endpoint. However, no postoperative activity restrictions were imposed and full mobilization was initiated on the day of surgery, rendering all ODI domains accessible from the first postoperative day onwards, as the ODI version 2.1a asks patients to select the statement that best describes them ‘today’. We therefore considered early postoperative ODI collection, analogue to several other studies, methodologically defensible, while interpreting these findings with appropriate caution (Gautschi et al., 2016; Chen et al., 2023; Yu et al., 2023; Li et al., 2024; Wang et al., 2025).

Finally, although restricting to baseline-engaged patients and to those being eligible for a 6-month follow-up reduces bias from insufficient observation time, observed retention may still be influenced by engagement propensity and unmeasured confounders. However, longitudinal interpretability was constrained by follow-up attrition. Future multicenter studies need to be performed to confirm these recovery and retention patterns across different care pathways and patient populations.

## 6 Conclusions

High-frequency, app-based PROM assessment enabled detailed characterization of distinct postoperative recovery patterns following full-endoscopic lumbar discectomy and decompression. Long-term PROM completeness was limited by attrition and differed between procedure types, with decompression associated with higher retention and a lower hazard of dropout than discectomy after adjustment for relevant covariates. Early postoperative PROM adherence was a strong independent predictor of sustained follow-up. These findings suggest that efforts to improve longitudinal outcome assessment after FESS should focus on strengthening patient engagement during the early postoperative period.

## Declaration of competing interest

The authors declare that they have no known competing financial interests or personal relationships that could have appeared to influence the work reported in this paper.
