# Supplementary material for: The first week matters: App-based PROM trajectories and follow-up retention after endoscopic lumbar surgery
Source: Brain Spine. 2026 May 10;6:106080. doi: 10.1016/j.bas.2026.106080 (PMC13188115; doi:10.1016/j.bas.2026.106080)
Supplement: Multimedia component 1 [file mmc1.docx]

Supplement Table S1. Excluded EELD-Cases in Discectomy cohort

App-based transmitted patient outcomes for VAS back pain, VAS leg pain, and ODI at the preoperative time point, first 7 postoperative days, 2 weeks, 3 and 6 months

|  |  | **Preop** | **Day 1** | **Day 2** | **Day 3** | **Day 4** | **Day 5** | **Day 6** | **Day 7** | **2 Wks** | **3 Mos** | **6 Mos** |
| --- | --- | --- | --- | --- | --- | --- | --- | --- | --- | --- | --- | --- |
| Discectomy |  | n = 276 | n = 257 | n = 253 | n = 249 | n = 252 | n = 241 | n = 245 | n = 258 | n = 271 | n = 276 | n = 182 |
|  | VAS back pain | 5.5 ± 2.8 | 3.7 ± 2.6; **< 0.001** | 3.5 ± 2.5; **< 0.001** | 3.0 ± 2.4; **< 0.001** | 3.0 ± 2.4; **< 0.001** | 2.8 ± 2.5; **< 0.001** | 2.8 ± 2.4; **< 0.001** | 2.7 ± 2.3; **< 0.001** | 2.4 ± 2.3; **< 0.001** | 2.2 ± 2.5; **< 0.001** | 2.4 ± 2.6; **< 0.001** |
|  | VAS leg pain | 6.2 ± 2.5 | 2.6 ± 2.8; **< 0.001** | 2.6 ± 2.7; **< 0.001** | 2.5 ± 2.5; **< 0.001** | 2.6 ± 2.6; **< 0.001** | 2.5 ± 2.6; **< 0.001** | 2.5 ± 2.5; **< 0.001** | 2.6 ± 2.6; **< 0.001** | 2.5 ± 2.6; **< 0.001** | 2.1 ± 2.7; **< 0.001** | 2.2 ± 2.7; **< 0.001** |
|  | ODI | 21.6 ± 9.4 | 19.3 ± 10.2; **< 0.001** | 19.2 ± 10.5; **< 0.001** | 18.1 ± 10.1; **< 0.001** | 18.6 ± 9.8; **< 0.001** | 17.5 ± 10.0; **< 0.001** | 17.0 ± 9.7; **< 0.001** | 16.9 ± 9.5; **< 0.001** | 15.1 ± 9.1; **< 0.001** | 9.2 ± 9.2; **< 0.001** | 9.1 ± 9.5; **< 0.001** |

Values are depicted as mean ± SD; p value. The p values refer to the reported value of the respective day compared with the preoperative value. Boldface type indicates statistical significance.

## Supplement Table S2.1-2. Between-group differences (ICELF, TELF, TE-LRD versus IELRD, LE-ULBD) for different timepoints from Linear Mixed Model with group × time interaction and random intercept for patient.

Supplement Table S2.1: Estimated between-group differences per timepoint

|  | **Preop** | **Day 1** | **Day 2** | **Day 3** | **Day 4** | **Day 5** | **Day 6** | **Day 7** | **2 Wks** | **3 Mos** | **6 Mos** |
| --- | --- | --- | --- | --- | --- | --- | --- | --- | --- | --- | --- |
| VAS back pain | 1.03 (0.13, 1.93) 0.0251 | -0.58 (-1.50, 0.34) 0.2163 | 0.19 (-0.72, 1.10) 0.6852 | -0.03 (-0.96, 0.90) 0.9488 | -0.05 (-0.98, 0.89) 0.9226 | -0.19 (-1.11, 0.74) 0.6930 | -0.22 (-1.18, 0.74) 0.6518 | -0.16 (-1.08, 0.76) 0.7336 | -0.41 (-1.31, 0.50) 0.3771 | -0.08 (-0.98, 0.81) 0.8536 | -0.16 (-1.18, 0.85) 0.7529 |
| VAS leg pain | 0.44 (-0.52, 1.39) 0.3707 | -1.15 (-2.13, -0.17) 0.0209 | -0.32 (-1.29, 0.65) 0.5126 | -0.53 (-1.52, 0.45) 0.2875 | -0.58 (-1.57, 0.41) 0.2513 | -0.45 (-1.43, 0.54) 0.3718 | -0.55 (-1.56, 0.47) 0.2924 | -0.68 (-1.66, 0.29) 0.1696 | -1.00 (-1.96, -0.04) 0.0409 | -0.12 (-1.07, 0.84) 0.8114 | -0.82 (-1.90, 0.27) 0.1403 |
| ODI | 2.04 (-1.42, 5.49) 0.2471 | 1.65 (-1.88, 5.19) 0.3579 | 3.71 (0.20, 7.22) 0.0386 | 2.96 (-0.59, 6.51) 0.1020 | 2.59 (-0.94, 6.13) 0.1503 | 0.78 (-2.76, 4.31) 0.6661 | 2.19 (-1.45, 5.84) 0.2371 | 1.44 (-2.07, 4.95) 0.4198 | 0.21 (-3.28, 3.70) 0.9067 | -0.03 (-3.49, 3.42) 0.9855 | -0.20 (-3.99, 3.60) 0.9184 |

Values are depicted as Δ with 95% CI; Holm-adjusted p.

Supplement Table S2.2: Global group × time interaction p-values:

| **Outcome** | **p Group Time** |
| --- | --- |
| VAS back pain | 0.101 |
| VAS leg pain | 0.147 |
| ODI | 0.244 |

## Supplement Table S3. Excluded Analgosedation-Cases in Discectomy and Decompression cohort

App-based transmitted patient outcomes for VAS back pain, VAS leg pain, and ODI at the preoperative time point, first 7 postoperative days, 2 weeks, 3 and 6 months

|  |  | **Preop** | **Day 1** | **Day 2** | **Day 3** | **Day 4** | **Day 5** | **Day 6** | **Day 7** | **2 Wks** | **3 Mos** | **6 Mos** |
| --- | --- | --- | --- | --- | --- | --- | --- | --- | --- | --- | --- | --- |
| Discectomy |  | n = 224 | n = 209 | n = 204 | n = 204 | n = 207 | n = 198 | n = 201 | n = 211 | n = 219 | n = 224 | n = 150 |
|  | VAS back pain | 5.3 ± 2.6 | 3.6 ± 2.5; **< 0.001** | 3.4 ± 2.4; **< 0.001** | 3.0 ± 2.4; **< 0.001** | 2.9 ± 2.3; **< 0.001** | 2.8 ± 2.3; **< 0.001** | 2.8 ± 2.2; **< 0.001** | 2.7 ± 2.2; **< 0.001** | 2.2 ± 2.1; **< 0.001** | 1.8 ± 2.1; **< 0.001** | 2.2 ± 2.4; **< 0.001** |
|  | VAS leg pain | 6.1 ± 2.5 | 2.3 ± 2.5; **< 0.001** | 2.3 ± 2.4; **< 0.001** | 2.4 ± 2.4; **< 0.001** | 2.4 ± 2.4; **< 0.001** | 2.4 ± 2.5; **< 0.001** | 2.4 ± 2.4; **< 0.001** | 2.4 ± 2.4; **< 0.001** | 2.2 ± 2.3; **< 0.001** | 1.6 ± 2.2; **< 0.001** | 1.8 ± 2.4; **< 0.001** |
|  | ODI | 21.7 ± 9.4 | 20.1 ± 10.1; **0.02966** | 20.1 ± 10.3; **0.03681** | 19.0 ± 9.9; **< 0.001** | 19.3 ± 9.7; **< 0.001** | 18.2 ± 9.8; **< 0.001** | 17.8 ± 9.5; **< 0.001** | 17.6 ± 9.5; **< 0.001** | 15.3 ± 9.0; **< 0.001** | 8.2 ± 8.6; **< 0.001** | 7.9 ± 8.6; **< 0.001** |
| Decompression |  | n = 196 | n = 182 | n = 189 | n = 184 | n = 181 | n = 181 | n = 180 | n = 190 | n = 195 | n = 196 | n = 139 |
|  | VAS back pain | 5.6 ± 2.5 | 4.2 ± 2.6; **< 0.001** | 4.3 ± 2.6; **< 0.001** | 3.9 ± 2.6; **< 0.001** | 3.5 ± 2.5; **< 0.001** | 3.4 ± 2.4; **< 0.001** | 3.2 ± 2.4; **< 0.001** | 3.0 ± 2.4; **< 0.001** | 2.7 ± 2.2; **< 0.001** | 2.6 ± 2.7; **< 0.001** | 2.8 ± 2.6; **< 0.001** |
|  | VAS leg pain | 6.0 ± 2.7 | 2.7 ± 2.7; **< 0.001** | 3.2 ± 2.8; **< 0.001** | 2.9 ± 2.7; **< 0.001** | 3.0 ± 2.7; **< 0.001** | 2.8 ± 2.6; **< 0.001** | 2.8 ± 2.5; **< 0.001** | 2.9 ± 2.6; **< 0.001** | 2.8 ± 2.5; **< 0.001** | 2.5 ± 2.8; **< 0.001** | 2.8 ± 2.6; **< 0.001** |
|  | ODI | 19.5 ± 8.4 | 21.4 ± 10.1; **0.01128** | 21.7 ± 10.6; **0.00311** | 21.4 ± 10.3; **0.01052** | 20.3 ± 10.3; 0.56329 | 19.7 ± 9.6; 0.99760 | 19.3 ± 9.4; 0.96732 | 19.3 ± 9.5; 0.99932 | 17.0 ± 9.2; **< 0.001** | 10.8 ± 9.0; **< 0.001** | 10.7 ± 9.0; **< 0.001** |

Values are depicted as mean ± SD; p value. The p values refer to the reported value of the respective day compared with the preoperative value. Boldface type indicates statistical significance.
